# Leadership in Digital Health Services: Protocol for a Concept Analysis

**DOI:** 10.2196/25495

**Published:** 2021-02-04

**Authors:** Elina Laukka, Tarja Pölkki, Tarja Heponiemi, Anu-Marja Kaihlanen, Outi Kanste

**Affiliations:** 1 Research Unit of Nursing Science and Health Management University of Oulu Oulu Finland; 2 Health and Social Service System Research Finnish Institute for Health and Welfare Helsinki Finland; 3 Medical Research Center Oulu University Hospital Oulu Finland

**Keywords:** health care, leadership, health services, concept analysis, telehealth

## Abstract

**Background:**

Due to the rapid digitalization of health care, leadership is becoming more complex. Leadership in digital health services is a term that has been used in the literature with various meanings. Conceptualization of leadership in digital health services is needed to deliver higher quality digital health services, update existing leadership practices, and advance research.

**Objective:**

The aim of this study is to outline a concept analysis that aims to clarify and define the concept of leadership in digital health services.

**Methods:**

The concept analysis will be performed using the Walker and Avant model, which involves eight steps: concept selection, determination of aims, identification of uses, determination of defining attributes, construction of a model case, construction of additional cases, identification of antecedents and consequences, and definition of empirical referents. A scoping literature search will be performed following the search protocol for scoping reviews by the Joanna Briggs Institute to identify all relevant literature on leadership in digital health services. Searches will be conducted in 6 scientific databases (CINAHL, MEDLINE, Scopus, ProQuest, Web of Science, and the Finnish database Medic), and unpublished studies and gray literature will be searched using Google Scholar, EBSCO Open Dissertations, and MedNar.

**Results:**

An initial limited search of MEDLINE was undertaken on October 19, 2020, resulting in 883 records. The results of the concept analysis will be submitted for publication by July 2021.

**Conclusions:**

A robust conceptualization of leadership in digital health services is needed to support research, leadership, and education. The concept analysis model of Walker and Avant will be used to meet this need. As leadership in digital health services appears to be an interprofessional and intersectoral collaboration, defining this concept may also facilitate collaboration between professionals and sectors. The concept analysis to be conducted will also expand our understanding of leadership in digital health services.

**International Registered Report Identifier (IRRID):**

PRR1-10.2196/25495

## Introduction

### Background

Several health care programs and reforms introduced in recent years have highlighted the importance of digitalization and health information technology for solving problems in modern health care [1,2]. This importance has been increased further by the COVID-19 pandemic [3]. Health care digitalization, also referred to as *digital transformation*, means that health services and systems are undergoing a transition whereby increasing numbers of these services and systems are becoming digitalized [4]. Although there is a rapidly growing body of research on health care digitalization [5], the viewpoint of health care leadership in digital health services has been de-emphasized [6-9]. Health care leaders have traditionally been responsible for clinical health services and their management [10]; however, now they are also responsible for developing and managing health care organizations’ health information technologies (HITs) [11-13]. Nurse leaders seem to be particularly active in planning the implementation of digital health services and bear the chief responsibility for its use [14]. This may be because nurses are often more involved in the early-stage implementation of digital transformation projects, whereas physicians join during later phases [15]. It has, therefore, been suggested that more research should focus on how to develop continuously evolving health care leadership that is well placed to cope with changes such as the current rapid digital transformation [16].

However, the literature provides little clarity as to what leadership in digital health services means or entails [17,18]. Researchers have used terms such as e-leadership [6,8] and virtual leadership [19] in reference to nurse leaders; however, their definitions are inconsistent. For example, e-leadership has several different definitions; among other things, it has been understood as a process of social influence that takes place in the context of an organization where work is supported by information and communication technology (ICT) [20]. In contrast, virtual leadership has been defined as leading remotely working teams [21]. In addition to these terms, expressions such as *physician leadership in eHealth* have been used [6]. The existence of several inconsistently defined concepts and meanings relating to leadership in digital health services makes it difficult to hold dialogs about the phenomenon. A recent scoping review by Strudwick et al [22] concluded that it is essential to understand the informatics competencies of nurse leaders because nurse leaders play key roles in all issues relating to technology in health services.

### What Is Known About Leadership in Digital Health Services?

Conceptualization of leadership and leadership-related issues is quite common in health care research. Previous concept analyses relating to health care leadership have focused on transformational leadership [24], staff nurse clinical leadership [25], nurse manager engagement [26], and nurse manager succession planning [27], among other things. As there has been limited research on leadership in digital health services [17,18], conceptualizing leadership in digital health services could provide important guidance for service development and future research. In this concept analysis, health care leadership will be considered to encompass both leading people and managing systems and structures [28]. The theory of transformational leadership, according to which leaders see change as an opportunity, has occasionally been linked to health care digitalization [17,29]. However, health care digitalization also seems to involve elements of management because implementing HITs requires decision making on various issues, including financial issues [30]. Health care organizations are among the most complex in society [31], and the increasing number of HITs makes them even more complex and challenging to manage for health care leaders [32-34].

Previous studies have mainly examined leadership in digital health services from the viewpoint of HIT implementation and adoption [7,13]. A recent scoping review by Laukka et al [13] found that roles adopted by health care leaders during HIT implementation include supporters, change managers, advocates, project managers, decision makers, facilitators, and champions. Another review by Ingebrigtsen et al [7] identified 7 leadership behaviors associated with successful outcomes in HIT adoption: communicating clearly about visions and goals, providing support, establishing a governance structure, establishing training, identifying and appointing champions, addressing work process change, and following up. These reviews synthesized leadership roles and behaviors important in HIT implementation and adoption. However, making the most of HIT also requires proper health care leadership in other processes [35]. For health care leaders, managing technology is also about the 3 *Ps*: people, processes, and (computer) programs [35]. The information age paradigm is transforming health care delivery and, in the process, may also shift leaders’ perspectives and shape their leadership responses [17].

Several studies have aimed to define the concept of e-leadership [36,37]; however, the definition has not been made in the context of health care, which is unique compared with other fields of businesses. According to Avolio and Kahai [36], “e-leadership takes place in a context where work is mediated by information technology.” Health care has lagged behind other areas of business in terms of the use of ICT [33,38,39], and efforts to implement HIT fail relatively frequently in health care settings [33,40]. This may be partly because of poor leadership in health care organizations [41,42].

To conclude, conceptualization of leadership in digital health services is needed to better understand how leadership can support health care digitalization and improve the likelihood of successful HIT implementation. For example, the integration of HIT is necessary for nurse leaders to lead effectively in the future [8]. Advancing digitalization and HIT implementation will enable the delivery of higher quality digital health services while also supporting health care professionals’ work related to HIT. In addition, a robust conceptualization will facilitate further research in this area and help reshape leadership models to establish digitalization as a core part of health care leaders’ core competency, thereby contributing to the provision of adequate education. Keijser et al [6] noted the importance of researching health care leadership in the context of digital health care and highlighted the importance of research in educating leaders.

### Objectives

Precise conceptualization of leadership in digital health services is needed to support health care leaders working on digitalization on the frontline and at middle and senior management levels, to help create better digital health services, to facilitate continuously evolving leadership, and to advance research. To this end, the concept of leadership in digital health services was analyzed using the concept analysis model of Walker and Avant [23]. The use of a protocol based on this model was expected to increase the quality of the final concept analysis.

## Methods

### This Study

#### Aim

The protocol outlines a concept analysis procedure designed to clarify and define the concept of leadership in digital health services.

#### Design

The concept analysis model of Walker and Avant [23], which has become one of the most influential concept analysis models in health care [43], will be used. The strength of the model by Walker and Avant [23] is that it provides a structural guideline. Walker and Avant [23] define concept analysis as the process of defining a concept carefully by understanding and examining its basic elements and underlying attributes. The 8 steps of their concept analysis procedure are described in the following sections.

#### Step 1: Selecting a Concept

The concept analysis process starts with the selection of a concept to be analyzed [23]—in this case, leadership in digital health services. Analysis of this concept is needed to establish an up-to-date definition and framework of leadership for the era of health care digitalization.

#### Step 2: Defining the Aim of the Analysis

The next step is to define the aims or purpose of the study [23]. Our specific aims in this study are to clarify the concept of leadership in digital health services and develop a theoretical definition of leadership in digital health services. Conceptualization of the construct of leadership in the context of digital health services is needed to better understand the phenomenon of health care digitalization, to guide future research, and to construct a modern leadership framework for health services.

#### Step 3: Identifying All Uses of the Concept

The third step involves first identifying all previous uses of the concept when collecting material for analysis [23]. In this study, diverse sources will be used to identify different definitions of leadership in digital health services. These sources will then be subjected to critical analysis to identify different definitions, descriptions, and applications of leadership in digital health services. The reported ways of using and describing leadership in digital health services will finally be recorded for future reference.

#### Step 4: Determining the Defining Attributes of the Concept

After identifying the different uses of the concept under investigation, the uses will be read through to find the characteristics that appear repeatedly to define the key attributes of the studied concept (in this case, leadership in digital health services). This process will generate *a cluster of attributes that are frequently associated with the concept* [23].

#### Step 5: Constructing a Model Case

This step involves developing one or more model cases to represent a real-life example of *the use of concept that includes all the critical attributes of the concept* [23]. This will be done using data extracted during the earlier phases.

#### Step 6: Constructing Additional Cases

After identifying at least one model case, additional cases relating to the concept under investigation will be identified. This is necessary because it will not be possible to complete the concept analysis if there are overlaps between the identified attributes or contradictions between the defining attributes and the model case [23]. The purpose of this step is to determine which characteristics or attributes best fit with the concept under study and to identify the attributes that define the concept [43]. The additional cases should include examples that are (1) related, (2) borderline, (3) contrary, (4) invented, and (5) illegitimate [23]. Related cases closely resemble the model case but can be seen to lack at least some of the defining attributes when examined closely. Borderline cases display some of the defining attributes but lack several others. These 2 cases help to clarify the concept and to show what it is not. A contrary case is one that is clearly not an instance of the concept, whereas an invented case is used to illustrate the essential features of a concept. Finally, an illegitimate case illustrates improper use of the concept [43].

#### Step 7: Identifying Antecedents and Consequences of the Concept

Antecedents and consequences will be identified in the penultimate step. Walker and Avant [23] defined antecedents and consequences, respectively, as events or incidents that occur before or as a result of the *occurrence of the concept.*

#### Step 8: Defining Empirical Referents

The final step of the concept analysis will be to integrate the critical attributes with real-world empirical referents. According to Walker and Avant [23], empirical referents are measurable ways to demonstrate the occurrence of the concept.

### Scoping Review

To identify all relevant literature on leadership in digital health services, a literature review will be conducted in accordance with the search protocol for scoping reviews by the Joanna Briggs Institute (JBI) [44]. Scoping reviews are useful for mapping the key concepts of a research topic and clarifying its working definitions and/or conceptual boundaries [45]. The search strategy used in a scoping review should be as comprehensive as possible [44]. This requirement aligns well with the principles of concept analysis, which call for the use of diverse sources to obtain varied definitions of the concept under investigation [43]. Unlike in a systematic review, quality assessment is not a necessary part of the scoping review process [44], and it is also not relevant in concept analysis [46,47]. Quality assessment is not needed in concept analysis because the data to be extracted relate to the definitions and attributes of leadership in digital health services, not the results of the study [46,47]. Therefore, all published uses of the concept under investigation are relevant, irrespective of the quality of the research in which they are used.

### Eligibility Criteria

The Population, Concept, and Context (PCC) framework will be applied when defining eligibility criteria for the scoping review [44]. Initial inclusion and exclusion criteria relating to the PCC of studies considered for inclusion in the scoping review are presented in Textbox 1.

**Eligibility criteria based on the Population, Concept, and Context framework for publications included in the scoping review.**

**Inclusion criteria**

- Population: Studies on health care leaders regardless of their management position or health care field
- Concept: Health care or service leadership
- Context: Digital health services

**Exclusion criteria**

- Population: Studies on leaders working solely with information technology management
- Concept: Not related to health care or service leadership
- Context: Health services with no digitalization of any kind

Both peer-reviewed publications and papers from gray literature will be included in the review. The studied population will consist of health care leaders or managers regardless of their management position and health care field. Leaders working solely with information technology management will be excluded because they are not responsible for clinical health services and management. Publications eligible for inclusion in the review will be those that somehow define or clarify the concept of leadership in the context of digital health services.

### Search Strategy

A 3-step search strategy [44] will be used to retrieve both published and unpublished studies. An initial limited search of MEDLINE was undertaken on October 19, 2020, as part of this study protocol, resulting in 883 papers (Table 1). Relevant papers were identified by analyzing their titles, abstracts, and index terms. MEDLINE was used in this preliminary search because its large database includes several papers relating to health care leadership; as such, the search was expected to provide a rough estimate of the number and availability of relevant papers. An information specialist was consulted when developing the initial search strategy and will be consulted about other search strategies as well. During the main concept analysis study, a search strategy using all the relevant identified keywords and index terms will be used for each information source to be searched. The reference lists of all included studies will also be screened to identify additional relevant studies.

The databases to be searched will include CINAHL, MEDLINE, Scopus, ProQuest, Web of Science, and the national Finnish database Medic. These databases collectively provide a comprehensive coverage of publications relating to health care leadership and digital health services. Searches for unpublished studies and gray literature will be conducted using Google Scholar, EBSCO Open Dissertations, and MedNar. Gray literature types eligible for inclusion will include editorials, opinion papers, and dissertations. Papers published in English, Finnish, and Swedish will be considered for inclusion. Only papers published between 2010 and the present (2020) will be considered for inclusion because the rapid digitalization of health services over the past decade [48] makes older studies less relevant to the current situation. Keywords to be used will be related to eHealth, information technology, digitalization, health care, health services, and leadership. Keywords will be truncated, where appropriate. In addition, index terms or headings such as Medical Subject Headings will be used in MEDLINE and CINAHL.

**Table 1:** Search strategy applied in MEDLINE using Medical Subject Headings terms and search terms with abstract, title, and keyword limitations. The search was undertaken on October 19, 2020.

Searches	Results, n
exp Telemedicine/	30,325
exp Leadership/	41,537
exp Telemedicine/ and exp Leadership/	85
(eHealth or e-health).tw^a^	4649
exp Telemedicine/ or (eHealth or e-health).tw^a^	33,067
(information technology or digital^*^).tw^a^	150,808
(health^*^ or medic^*^ or nursing^*^).tw^a^	4,352,122
(information technology or digital*).tw^a^ and (health* or medic* or nursing*).tw^a^	36,072
exp Telemedicine/ or (eHealth or e-health).tw^a^ or (information technology“ or digital*).tw^a^ and (health* or medic* or nursing*).tw^a^	66,516
“leader^*^”.tw^a^	76,359
exp Leadership/ or “leader*”.tw^a^	97,028
Telemedicine/ or (eHealth or e-health).tw^a^ or (information technology or digital*).tw^a^ and (health* or medic* or nursing*).tw^a^ and exp Leadership/ or “leader*”.tw^a^	1217
limit Telemedicine/ or (eHealth or e-health).tw^a^ or (information technology or digital*).tw^a^ and (health* or medic* or nursing*).tw^a^ and exp Leadership/ or “leader*”.tw^a^ to y=“2010-Current*”*	883

^a^Text word terms are searched from the titles, abstracts, and keywords.

### Study Selection

All citations identified by implementing the search strategy described earlier will be collated and uploaded into the Covidence systematic review systematic software package (v2422), which will also be used to remove duplicates. Titles and abstracts will then be screened by two team members independently using the inclusion or exclusion criteria. For papers without abstracts, the full text will be retrieved. After title and abstract screening, the potentially relevant studies will be retrieved in full. Two independent team members will assess these studies in detail and evaluate their suitability based on the inclusion criteria. Reasons for exclusion will be reported for studies that do not satisfy the inclusion criteria. Any disagreements at any stage of the study selection process will be resolved by discussion or by asking the opinion of a third team member. The results of the search will be reported in the final study and will be presented in a PRISMA (Preferred Reporting Items for Systematic Reviews and Meta-Analyses) flow diagram [49]. All search methods, strategies, and sources will be described or named in the final report and will be replicable.

### Data Extraction and Synthesis

The extracted data will include definitions of leadership in digital health services; its key domains; the setting and population of the study described in the paper; and data needed for the 8-step concept analysis, such as attributes, antecedents, and consequences [23]. Two researchers will participate in data extraction.

### Ethical Considerations

As concept analyses use only secondary publicly available data from primary research studies and gray literature, no research ethics approval will be needed.

### Validity and Rigor

Several activities will be performed to enhance the study’s validity and rigor, including the following:

1. Method: The scoping review, which will identify all relevant literature on leadership in digital health services, will be conducted following the JBI guidelines [44].
2. Search: An information specialist with expertise in health sciences and management research will be consulted when developing the search strategy to increase credibility. In addition, several databases and gray literature sources will be included to ensure the richness of the data.
3. Screening, data extraction, and synthesis: Each of the previously mentioned phases will be conducted independently by 2 team members. Having 2 independent team members to select papers, extract data, and conduct synthesis will enhance reliability.
4. All members of the research team will repeatedly evaluate the manuscript during meetings that will be held as the process progresses.

## Results

The search for the relevant studies was performed on November 31, 2020, resulting in 2861 studies after duplicates were removed. The screening of the studies will be completed by the end of January 2021. We expect to begin other phases of concept analysis in February 2021. The concept analysis is anticipated to be ready for submission by July 2021.

## Discussion

### Principal Findings

In recent years, leadership in digital health services has been scrutinized in relation to issues including HIT adoption and implementation [7,13,50], informatics competence [51,52], and virtual teams [6,21]. Previous studies suggest that there is no consistent treatment of different elements of digitalization (eg, implementation, informatics competence) within leadership in health services and that current approaches to leadership in digital health services are therefore fragmentary and incomplete.

The literature indicates that all health care leaders, regardless of their management position, are involved in health care digitalization [9,13-15] and that nurse leaders play a particularly important role in the early use of HIT [15]. The roles of frontline nurse or physician leaders seem to be essential in supporting health care professionals in the use of HIT [13]. Nurses and physician leaders working in middle management also play an important role in implementing HIT [14], and senior nurses and physician managers seem to be responsible for making decisions about obtaining new HIT [30,53]. Collaborations between nurse leaders and chief information officers have also been scrutinized [50,54]. Overall, leadership in digital health services seems to require interprofessional and intersectoral collaboration involving working together with other health care leaders, chief information managers, health care professionals, research and educational centers, and HIT vendors [14,50,54,55].

Glaser [56] suggested that HIT implementation failures are often because of the *actions and inactions of senior leadership*. This may be because health care leaders might have insufficient informatics skills. For example, according to Sharpp et al [57], some nurse leaders are inexperienced users of ICT. Several studies have proposed that health care leaders have not received enough, or any, education on informatics [13,29,58]. HIT, thus, seems to be a *black box* for some health care leaders, and this issue should be examined more thoroughly [30].

### Limitations

The preliminary search of MEDLINE conducted while developing this study protocol retrieved many potentially relevant papers. Therefore, the search strategy (especially for the largest databases) will involve only title and abstract searches to ensure that the review balances feasibility with comprehensiveness. Limiting the searches to titles and abstracts may cause some relevant studies to be excluded from the concept analysis. However, title limitation appears to be quite common in reviews focusing on health care digitalization [13,59]. The concept analysis model by Walker and Avant [23] provides the analyst 8 steps to guide their analysis. However, despite these 8 steps, the more detailed analysis has been left to the analyst to figure out individually [43].

### Conclusions

There is a clear need to conceptualize leadership in digital health services because leadership in health services seems to be incoherent, providing no consistent perspective on the phenomenon. In addition, this study shows that the concept analysis model of Walker and Avant [23] is suitable for conceptualizing leadership in digital health services. Such a concept analysis could be beneficial in several ways. First, it could help guide research on and modelling of leadership within health care studies. Second, providing a clear definition of leadership in digital health services could guide health care leaders and managers in their work, facilitate interprofessional and multisectoral collaboration, and advance clinical practice, especially in relation to digitalization. Third, conceptualization could be used to guide the training of health care leaders to help them better meet current and future challenges relating to health care digitalization.

Our evaluation of the need to conceptualize leadership in digital health services made it clear that in addition to providing a basis for further research, defining and clarifying the concept of leadership in digital health services could facilitate the development of higher quality digital health services by actualizing the roles and responsibilities of leaders in digitalized health care. A clear definition may also help educational and health care organizations to provide better education and training for health care leaders in ICT, which would, in turn, support the digitalization of health care.

## Abbreviations

HIT: health information technology
ICT: information and communication technology
JBI: Joanna Briggs Institute
PCC: Population, Concept, and Context
